# Polysomnography findings in sleep-related eating disorder: a systematic review and case report

**DOI:** 10.3389/fpsyt.2023.1139670

**Published:** 2023-05-10

**Authors:** Bartlomiej Blaszczyk, Tomasz Wieczorek, Monika Michalek-Zrabkowska, Mieszko Wieckiewicz, Grzegorz Mazur, Helena Martynowicz

**Affiliations:** ^1^Student Research Club No K133, Faculty of Medicine, Wroclaw Medical University, Wroclaw, Poland; ^2^Department and Clinic of Psychiatry, Wroclaw Medical University, Wroclaw, Poland; ^3^Department and Clinic of Internal Medicine, Occupational Diseases, Hypertension and Clinical Oncology, Wroclaw Medical University, Wroclaw, Poland; ^4^Department of Experimental Dentistry, Wroclaw Medical University, Wroclaw, Poland

**Keywords:** sleep-related eating disorder, SRED, polysomnography, PSG, nocturnal eating, parasomnia

## Abstract

**Background:**

Sleep-related eating disorder (SRED) consists of recurrent episodes of uncontrolled, involuntary eating and drinking 1–3 h after falling asleep with partial or full unconsciousness. This condition is diagnosed based on interviews with the patients affected and the diagnostic criteria of the International Classification of Sleep Disorders. However, polysomnography (PSG) is not necessary to confirm this disease. This systematic review aims to evaluate the findings of PSG in SRED patients.

**Methods:**

For this systematic review, PubMed, Embase, and Scopus databases were searched in February 2023, which resulted in 219 records. After removing duplicates, the articles that included the presentation of PSG results of SRED patients in English were selected. In addition, only original studies were considered. The risk of bias by using case reports and descriptive studies was assessed using the Joanna Briggs Institute critical appraisal tools and the Risk of Bias In Non-randomized Studies of Interventions (ROBINS-I) tool. Furthermore, a case report of a 66-year-old woman with SRED was included.

**Results:**

A total of 15 papers were selected for further analysis, of which 7 were descriptive studies, 6 were case reports, and 2 were observational studies. The risk of bias in the majority of the studies was moderate or high. Unexpectedly, if the eating episode occurred during PSG, in most cases it was not observed during deep sleep (the N3 sleep stage). Moreover, studies did not report significant deviations in the sleep parameters measured using PSG. Among SRED patients, the prevalence of sleepwalking was much higher than the general population. Our case report presented a potentially life-threatening episode of holding an apple in the mouth that might result in choking, which was captured using PSG.

**Conclusion:**

Polysomnography is not necessary for the diagnosis of SRED. However, it could facilitate the diagnosis and differentiation of SRED from other eating disorders. PSG also has limitations in capturing eating episodes and in addition, its cost effectiveness should be considered during the diagnostic process. More studies into the pathophysiology of SRED are needed because classifying SRED as non-rapid eye movement parasomnias can be inappropriate as it does not always occur during deep sleep.

## 1. Introduction

Sleep-related eating disorder (SRED) consists of recurrent episodes of uncontrolled, involuntary eating and drinking 1–3 h after falling asleep with partial or full unconsciousness (1). After arousal from non-rapid eye movement sleep (NREM), patients often consume high-calorie food products but also inedible and toxic food (2). In addition, cases of life-threatening or dangerous situations of eating food have been described in the literature (3). SRED is an example of NREM parasomnias, which also includes sleepwalking, confusional arousals, and sleep terrors (4). The presence of disease is estimated in about 5% of the general population (5), especially occurring in women in their mid-20 s (6). Other sleep diseases are often associated with SRED: sleepwalking called formerly somnambulism, obstructive sleep apnea (OSA), narcolepsy, periodic limb movements syndrome (PLMS) or restless leg syndrome (RLS) (2). The pathophysiology of SRED is not completely understood, but it is probably associated with the disability of the brain's reward system activation (7). Predisposing factors for SRED include female gender, mental stress, depression, and genetic factors (2), but the most common is drug-induced SRED by drugs such as zolpidem, serotonin norepinephrine reuptake inhibitors, and quetiapine (8), which is called secondary SRED. According to the International Classification of Sleep Disorders, Third Edition (ICSD-3), the condition under concern should meet all the following criteria from Table 1 to be diagnosed as SRED (9).

**Table 1:** The diagnostic criteria for sleep-related eating disorder (SRED) and for nocturnal eating syndrome (NES).

**Diagnostic criteria for sleep-related eating disorder (SRED) according to the international classification of sleep disorders, third edition**	**Diagnostic criteria for nocturnal eating syndrome (NES) according to the international symposiums on night eating**
1. Recurrent episodes of eating following arousal from sleep or during the main sleep stages	1. Evening or nocturnal hyperphagia, characterized by eating at least >25% of the total daily calories or at least two episodes of nocturnal food ingestion per week
2. Coexisting with at least one of the following condition in association with the recurrent episodes of involuntary eating: - eating of toxic/inedible food or substances; - adverse effects of nocturnal eating on health; - sleep-related injurious behaviors related to capturing/cooking the food	2. Coexisting to nocturnal eating at least three of five below features: - lack of desire to eat in the morning (morning anorexia); - strong urge to eat between dinner and sleep initiation or in night; - problems with sleep initiation or sleep insomnia at least four times per week; - persistent belief that eating allow to initiate or return to sleep; - decreased mood or depression during the night
3. There is partial or complete loss of consciousness, inability to regain awareness on eating episodes	3. Full awareness during nocturnal eating episodes, complete ability to remind about ingestion circumstances
4. Exclusion of other causes/disorders	4. Exclusion of other causes/disorders
	5. Significant distress or daily impairment in functioning
	6. Episodes maintain at least 3 months

Importantly, a polysomnography (PSG) examination is not required for the diagnosis of SRED although it is a “gold standard” procedure nowadays to detect sleep disorders. PSG includes electroencephalography, electromyography, electrocardiography, recording of body and limb movements, airflow measurement, oxygen saturation recording, recording of chest and abdomen wall movements, and video monitoring; therefore, it provides qualitative and quantitative parameters for abnormalities during sleep (10). In addition, the level of awareness of SRED episodes can be measured (2). Full consciousness during eating episodes and eating at least >25% of the total daily calories at night are characterized by a similar condition to SRED called Nocturnal Eating Syndrome (NES) (11). Despite obvious mentioned differences between these 2 conditions and other features of NES from Table 1 (12), sometimes their symptoms may overlap (13). Additionally, previously mentioned sleep disorders may coexist with SRED (14); thus, PSG should be performed to exclude any aforementioned disturbances. Many previous studies have reported only suspected side effects of drugs or patients' symptoms, such as suspicious behavior of uncontrolled nocturnal eating, without confirming any additional sleep disturbances. However, PSG findings, especially with video recording, have revealed the characteristic behavior of parasomnia in difficult cases and helped distinguish these conditions from SRED to treat the patients appropriately (4).

Given these circumstances, the primary objective of this review was to evaluate the existing results of PSG performed in patients with SRED. This review also included a case report of a patient admitted to the Sleep Laboratory at the Wroclaw Medical University due to fatigue, daytime drowsiness, snoring, obesity, and several nocturnal eating episodes per month, which were observed using PSG and not commonly found in the literature of eating during sleep.

## 2. Methods

### 2.1. Search strategy and data sources

This systematic review, which was not a registered review, was designed following the Preferred Reporting Items for Systematic Reviews and Meta-Analyses 2020 (PRISMA 2020) Checklist (15, 16). Three databases, namely Embase, PubMed, and Scopus, were searched with appropriate filters on February 8, 2023. All studies without limited time frame were included. The search term used was “sleep related eating disorder” OR “sleep-related eating disorders” OR “SRED” AND “polysomnography” OR “PSG” OR “polysomnographic” OR “examination results”. Two authors (BB and HM) separately performed the search, and the results were compared. After removing duplications, the remaining records were screened based on their title and abstract. Then, according to the exclusion criteria presented in Section 2.2 and PRISMA 2020 guidelines, studies that did not meet the criteria were excluded. To confirm eligibility, appropriate full-text papers were read by two authors (BB and HM). In case of disagreements, the third researcher (MM-Z) resolved them via discussion.

### 2.2. Inclusion and exclusion criteria

The inclusion criteria of the studies were as follows: full-text papers, published in English and presentation of SRED patients with video-PSG (v-PSG) results. All patients' age, gender, and comorbidity disorders were considered. The difference between primary SRED and drug-induced SRED was not considered. PSG results could be presented as analytical or descriptive outcomes. The following were the exclusion criteria: non-English papers, lack of access to the records and type of studies as review, systematic review, book chapters, letters to the editor, commentaries, conference abstracts and articles where PSG results of SRED were combined with the nocturnal eating syndrome (NES). Furthermore, if an article had insufficient data to assess eligibility, they were excluded.

### 2.3. Data extraction and assessment of bias risk

After selecting the studies that fulfilled the above criteria, the data from the selected studies were extracted. Two reviewers (BB and HM) created a table containing the primary characteristics of the articles, such as author, type, number of participants, age and gender of patients, and their actual concomitant sleep disorders. In addition, episodes of nocturnal eating during PSG and polysomnographic parameters were also obtained from the selected studies.

Finally, the risk of bias in the included studies was assessed. Due to the diverse variety of article types, the Joanna Briggs Institute (JBI) critical appraisal tools for case reports and qualitative (descriptive) studies were used (17). For the remaining findings, the Risk of Bias In Non-randomized Studies of Interventions (ROBINS-I) tools for non-randomized studies were used (18).

To evaluate the methodological quality, in accordance with the JBI checklist, 8 questions were answered for case reports, and 10 questions for qualitative studies. The possible answers were “yes”, “no”, “unclear”, or “not applicable”. A low risk of bias was assumed when the answer “yes” was observed at least 7 times for case reports and 8 times from 10 categories for descriptive studies. A high risk of bias was assumed when the answer “yes” was observed less than 5 times for case presentations and less than 6 times for qualitative records. A moderate grade was observed between the two mentioned ranges.

The 7 domains of each study were checked using ROBINS-I to assess the quality of the studies. The risk of bias was graded as “low”, “moderate”, “serious”, “critical”, or “no information”. The risk of bias was considered “serious” or “critical” if the article obtained “serious” or “critical” ratings at least in one domain. Studies that achieved “low” in all categories were considered having a low risk of bias, and studies without any “serious” and “critical” ratings were considered having a moderate risk of bias. Assessments of the risk of bias were always conducted separately by two researchers (BB and HM), who reached the final result via discussion.

## 3. Results

### 3.1. Study selection

**Figure 1:**
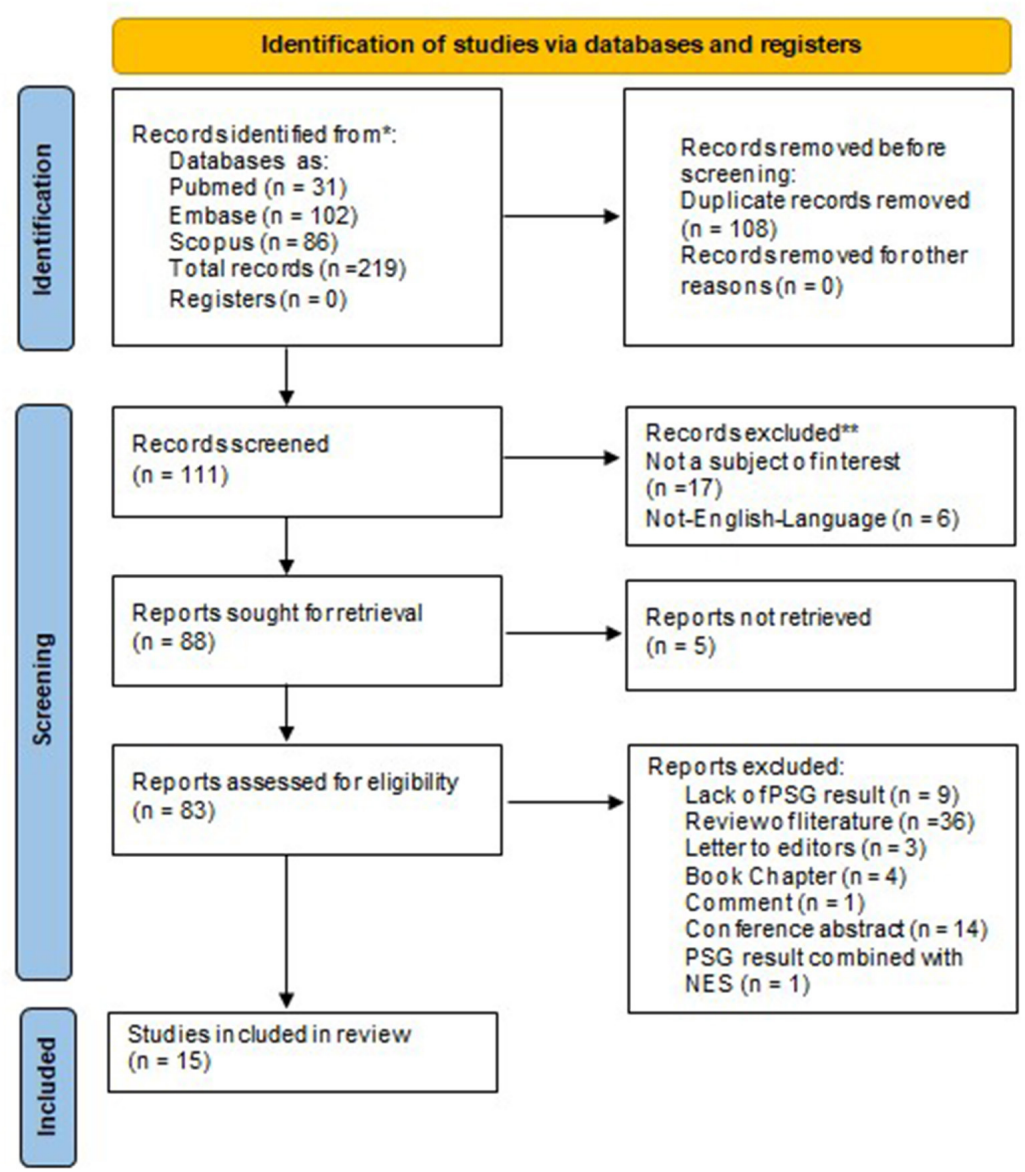
Preferred reporting items for systematic reviews and meta-analyses 2020 flow diagram. PSG, polysomnography.

The search resulted in 219 studies, 102 in Embase, 31 in PubMed, and 86 in Scopus, of which 108 were duplicates and hence removed. Among the remaining 111 studies, 17 did not belong to the subject of interest, 6 were not published in English, and 5 could not be retrieved. Among 83 full-text papers, 36 studies were reviews of literature, 3 were letters to the editor, 4 were book chapters, 14 were conference abstract and 1 was commentary. In addition, the PSG examination was not performed in 9 articles and in 1 article authors combined the PSG result of SRED with NES. Hence, only 15 manuscripts were selected for further analysis and evaluation (7, 13, 14, 19–30). A detailed description of the studies selection process is presented in Figure 1. The primary characteristics of studies with PSG examination in patients with SRED are summarized in Table 2.

**Table 2:** The major characteristics of studies containing polysomnography examination in patients with sleep-related eating disorder.

**Study**	**Type of study**	**Patient group**	**Age**	**Actual concomitant sleep disorders**	**Episodes of nocturnal eating during PSG**	**Polysomnographic parameters**
Yeh & Schenck (21)	Case report	1 male	29 years old	Not reported	4 episodes in the N2 stage	TST: 240 min (N1: 2.4%, N2: 50.6%, N3: 13.1%, REM: 8.7%), SE: Not reported, AI: Not report, AHI: Not reported, PLMSI: Not reported, WASO: Not reported, REM latency: Not reported
Wallace et al. (22)	Case report	1 male	42 years old	Narcolepsy–cataplexy, OSA	Not reported	TST: 513.0 min (N1: 4%, N2: 41.4%, N3: 49.3%, REM: 5.4%), SE: 95.2%, AI: Not reported, AHI: Not reported, PLMSI: Not reported, WASO: 1.0 min, REM latency: 418.5 min
Perogamvros et al. (7)	Case series	2 males	45 years old	Sleepwalking	Not reported	TST: 438 min (N1: 11%, N2: 66%, N3: 9%, REM: 14%), SE: 79%, AI: Not reported, AHI: 13.4/h, PLMSI: 0/h, WASO: Not reported, REM latency: 273 min
			29 years old	Sleepwalking	Not reported	TST: 440 min (N1: 8%, N2: 46%, N3: 25%, REM: 21.5%), SE: 94.6%, AI: Not reported, AHI: 13.4/h, PLMSI: 1/h, WASO: Not reported, REM latency: 108 min
Nzwalo et al. (20)	Case report	1 female	53 years old	Not reported	1 episode in the N1 stage	TST: 424 min (N1: Not reported, N2: Not reported, N3: Not reported, REM: Not reported), SE: 81.9%, AI: Not reported, AHI: Not reported, PLMSI: 0/h, WASO: Not reported, REM latency: Not reported
Varghese et al. (14)	Case report	2 males	35 years old	Not reported	Not reported	TST: 416 min (N1: 9%, N2: 54%, N3: 14.5%, REM: 22.5%), SE: 85%, AI: 11/h, AHI: 2.8/h, PLMSI: 0/h, WASO: 52 min, REM latency: Not reported
			37 years old	Not reported	Not reported	TST: 414 min (N1: 3%, N2: 49%, N3: 20%, REM: 28%), SE: 92%, AI: 20/h, AHI: 1/h, PLMSI: 0/h, WASO: 52 min, REM latency: Not reported
Ghosh et al. (19)	Case report	1 male	9 years old	Not reported	Not reported	TST: 210.5 min (N1: 2.6%, N2: 37.2%, N3: 44.2%, REM: 15.9%), SE: 47%, AI: 4/h, AHI: 0/h, PLMSI: 0/h, WASO: 191.0 min, REM latency: 341.5 min
Schenck et al. (26)	Descriptive	14 females and 5 samales	37.4 ± 9.1 years old	16 Sleepwalking, 2 PLMS, 2 Narcolepsy	Not reported	TST: 447.2 ± 91.5 min (N1: 6.2 ± 4.4%, N2: 48.6 ± 7.6%, N3: 23.4 ± 5.5%, REM: 21.8 ± 5.9%), SE: 85.6 ± 17.7 %, AI: Not reported, AHI: Not reported, PLMSI: 0/h, WASO: Not reported, REM latency: 76.2 ± 28.3 min
Schenck et al. (27)	Descriptive	11 females and 8 males	40.1 ± 10.6 years old	8 Sleepwalking, 3 RLS/PLMS, 4 OSA,	4 episodes in the N1 and N2 stages, 2 in the N3 stage, 1 in REM	TST: Not reported (N1: Not reported, N2: Not reported, N3: Not reported, REM: Not reported), SE: Not reported, AI: 16/h in 1 case, AHI: Not reported, PLMSI: 49/h in 1 case, WASO: Not reported, REM latency: Not reported
Winkelman et al. (24)	Descriptive	19 females and 4 males	21.6 ± 10.9 years	11 Sleepwalking,	3 episodes in N3 stage, 1 in N2 stage, 1 in REM	TST: 392.5 ± 51.9 min (N1: 11.0 ± 4.0%, N2: 48.9 ± 6.1%, N3: 21.6 ± 10.0%, REM: 20.4 ± 6.6%), SE: 88.3 ± 8.8 %, AI: 18.3 ± 5.8/h, AHI: 4.6 ± 8.3/h, PLMSI: 2.4 ± 4.7/h, WASO: Not reported, REM latency: 115.1 ± 60.1 min
						TST: 352.4 ± 52.7 min (N1: 16.5 ± 10.1%, N2: 55.0 ± 15.6%, N3: 12.5 ± 10.0%, REM: 12.4 ± 9.9%), SE: 80.2 ± 8.6 %, AI: 22.0 ± 12.6/h, AHI: 10.8 ± 25.5/h, PLMSI: 9.2 ± 12.1/h, WASO: Not reported, REM latency: 142.7 ± 91.5 min
Vetrugno et al. (25)	Descriptive	21 females and 14 males	44 ± 12.7 years old	1 Sleepwalking, 8 RLS, 4 PLMS	19 episodes in the N2 stage, 13 in the N3 stage, 1 in REM, 2 in wakefulness	TST: 426.9 ± 96.7 min (N1/N2: 60.5 ± 8.2%, N3: 17.9 ± 8.1%, REM: 21.6 ± 5.8%), SE: 76.2 ± 18.5%, AI: 19 ± 11.3/h, AHI: Not reported, PLMSI: 26.7 ± 18.7, WASO: Not reported, REM latency: 108.5 ± 72 min
Santin et al. (28)	Descriptive	23 females and 11 males	39.0 ± 13.8 years old	9 OSA, 16 RLS, 4 Sleepwalking, 1 sleep terror	4 episodes in the N2 and N3 stages	TST: Not reported (N1: Not reported, N2: Not reported, N3: Not reported, REM: Not reported), SE: Not reported, AHI: Not reported, AI: average 48.4/h, PLMSI: Not reported, WASO: Not reported, REM latency: Not reported
Vinai et al. (13)	Descriptive	2 females and 4 males	44.6 ± 12.93 years old	Not reported	2.2 ± 1.9 episodes	TST: 354.0 ± 80.1 min (N1/N2: 56.0 ± 6.0%, N3: 23.0 ± 6.2%, REM: 19.2 ± 3.8%), SE: 82.1 ± 16.9%, AI: 14.4 ± 7.5/h, AHI: Not reported, PLMSI: Not reported, WASO: Not reported, REM latency: 135.8 ± 77.0 min
Drakatos et al. (23)	Descriptive	2 females and 5 males	43.8 ± 18.2 years old	2 OSA, 1 PLMS, 2 OSA and/or PLMS	Not reported	TST: 391.3 ± 63.4 min (N1:8.4 ± 3.5%, N2: 52.7 ± 12.4%, N3: 25.8 ± 7.1%, REM: 17.0 ± 5.4%), SE: 82.3 ± 10.4%, AI: 20.3 ± 7.2/h, AHI: 6.5 ± 10.9/h, PLMSI: 12.7 ± 24.4/h, WASO: 58.3 ± 37.3 min, REM latency: Not reported
Komada et al. (29)	Cohort study	20 females and 10 males	32.2 ± 0.5 years old	Not reported	3 episodes in wakefulness, 12 in the N2 stage, 1 in the N3 stage	TST: 449.0 ± 68.2 min (N1: 10.2 ± 4.6%, N2: 52.9 ± 11.8%, N3: 6.8 ± 5.4%, REM: 18.9 ± 5.0%), SE: 86.7 ± 10.8%, AI: 14.1 ± 5.0 /h, AHI: 2.4 ± 3.5/h, PLMSI: 0/h, WASO: Not reported, REM latency: 90.9 ± 72.7 min
		5 females and 5 males	45.3 ± 15.0 years old	Not reported		TST: 460.4 ± 113.5 min (N1: 7.4 ± 3.0%, N2: 62.5 ± 5.7%, N3: 1.4 ± 1.3%, REM: 15.2 ± 6.5%), SE: 82.2 ± 12.7%, AI: 10.9 ± 2.9/h, AHI: 1.3 ± 0.7/h, PLMSI: 1.2 ± 2.8/h, WASO: Not reported, REM latency: 162.3 ± 155.1 min
Brion et al. (30)	Case–control study	12 females and 3 males	47.5 ± 15.1 years old	4 RLS, 7 Sleepwalking	9 episodes in the N2 stage, 6 in the N3 stage	TST: 426.9 ± 96.7 min (N1: 6.7 ± 4.6%,N2: 49.2 ± 9.7%, N3: 25.9 ± 10.7%, REM: 18.5 ± 7.9%), SE: 85.2 ± 12.9%, AI: 18.4 ± 13.9/h, AHI: 7.6 ± 13.7, PLMSI: 9.9 ± 19.6/h, WASO: Not reported, REM latency: 135.8 ± 77.0 min

RLS, restless legs syndrome; OSA, obstructive sleep apnea; PLMSI, Periodic Limb Movement in Sleep Index; TST, total sleep time; N1 stage sleep; N2 stage sleep; N3 stage sleep; REM, rapid eye movement; SE, sleep efficiency; AI, Arousal Index; AHI, Apnea–Hypopnea Index; WASO, wake after onset sleep; min, minutes.

### 3.2. Risk of bias in studies

The risk of bias was evaluated for the 6 case reports included in this review. Five studies (7, 14, 19–21) was evaluated on 5 to 6 answers of “yes” using the JBI checklist; therefore, their overall risk of bias was considered moderate. One of them showed a high risk of bias as it achieved “yes” less than 5 times (22). The steps of the assessment for case reports are presented in detail in Supplementary Table 3. Among descriptive articles, a moderate risk of bias was observed in 6 studies as 3 study achieved “yes” 6 times and 3 achieved the same 7 times (23–28). 1 study was estimated as having a low risk of bias as it achieved “yes” 8 times (13). The detailed steps of the assessment for descriptive studies are presented in Supplementary Table 4. The ROBINS-I tool was used to evaluate 2 studies. Komada et al. (29) had missing data; therefore, it was considered having a serious risk of bias. Brion et al. (30) achieved low or moderate scores in each domain, and according to the relevant criteria, the overall quality was moderate. This evaluation is presented in Supplementary Table 5.

### 3.3. Study characteristics

#### 3.3.1. The general information

All the included studies presented PSG results of patients with SRED who were examined for the reported symptoms (7, 14, 19–22) for the following reasons: detecting the polysomnographic features of this syndrome (13, 23, 24, 29, 30), differentiating SRED from other diseases characterized by nocturnal eating (25–27), and explaining the pathophysiology of SRED (28). The majority of the included records were descriptive studies (13, 23–28), as well as case reports (14, 19–22), the rest of them were 1 case series (7), 1 cohort study (29) and 1 case control study (30). In the studies presenting individual cases, i.e., in case reports and case series, the exact age and polysomnographic parameters were provided. However, if a study included a large number of patients, the average values were presented with appropriate standard deviations (13, 23–30). In eight studies, the majority of the participants were female (20, 24–30). The total number of presented patients in included studies were 206 people. The youngest patient in this review was 9 years old (19), whereas the oldest one aged 70 years (30).

#### 3.3.2. Concomitant sleep disorders

Coexisting sleep disorders such as NREM parasomnias and those predisposing to SRED were also considered (14). Single disorders were diagnosed, mainly sleepwalking in Perogamvros et al. (7) and Winkelman (24). It is worth mentioning that a single patient may have several sleep pathologies (22), e.g., narcolepsy–catalepsy and obstructive sleep apnea. In studies with a large sample size, only the number of disorders in the group was reported, without specifying whether these disorders affected **one** or more patients. For example, OSA was observed 18 times (22, 23, 27, 28), RLS 31 times (25, 27, 28, 30), PLMS 12 times (23, 25–27), sleepwalking 49 times (7, 24–28, 30), and sleep terrors 1 time (28). Only in 6 studies, no current sleep disturbances were reported (13, 14, 19–21, 29). Therefore, the prevalence of mentioned sleep disorders among presented patients were: 8.73% of OSA, 15.0% of RLS, 5.83% of PLMS, 23.9% of sleepwalking and 0.5% was sleep terrors.

#### 3.3.3. Polysomnography findings

Due to the use of v-PSG, which is a good diagnostic software, and the consumption of food during the study, nine studies observed sleep arousals for food intake, which occurred once or multiple times (13, 20, 21, 24, 25, 27–30). Episodes of eating were observed 5 times from N1 sleep stage (5.26% of total episode eating) (20, 27), 53 times from N2 sleep stage (55.81%) (21, 24, 25, 27–30), 29 times from deep sleep – N3 sleep stage (30.51%) (24, 25, 27–30), 3 times in rapid eye movement (REM) (3.16%) (24, 25, 27) and 5 times during wakefulness (5.26%) (25, 29). The last characteristic considered in this systematic review was PSG results, which consisted of the following parameters: total sleep time (TST) presented in minutes and in percentages (%); stage of sleep, N1, N2, N3, REM; sleep efficiency (SE) in percentages (%); Arousal Index (AI) in events per hours (e/h); Apnea–Hypopnea Index (AHI) in e/h; Periodic Limb Movements During Sleep Index (PLMSI) in e/h; wake after sleep onset (WASO) in minutes; and REM latency in minutes. When a study did not include these parameters, it was indicated as “Not reported” in the table. The results can be divided according to the approach of presentation. In 1 study, only 1 selected parameter was presented (27, 28), and in 7 studies that were not case studies/series, the 1 were presented as means with standard deviations (13, 23–26, 29, 30). The remaining studies had complete PSG results. These results are presented in Table 2.

## 4. Case presentation

A 66-year-old Caucasian woman with multiple disease conditions, namely hypertension, type 2 diabetes, overweight, atherosclerosis, osteoporosis, hypercholesterolemia, post-stroke conditions, insomnia, benzodiazepine drug dependence, visual impairment, and major depressive disorder (MDD), was admitted to the Sleep Laboratory in the Department of Internal Medicine, Occupational Diseases, Hypertension and Clinical Oncology, Wroclaw Medical University, Poland. She had a long history of various diseases sleep disorders, including problems with sleep, daytime sleepiness, and snoring. Informed consent was obtained from the patient for the publication of her case.

### 4.1. Description of sleep problems

During the detailed medical interview, she reported eating episodes at night, usually several times a month. These episodes had been occurring for the past 2 years, and the patient did not report any parasomnia episodes in the earlier years. She remembered some of these events, whereas some were covered by partial or complete amnesia. She often used products with a high glycemic index, i.e., fruit and sweets. She had never eaten an inedible product. Her husband, who witnessed her eating at night, informed her about some of the episodes. In the morning, she often found a mess in her kitchen, such as open cabinets, torn food packages, etc.

### 4.2. Laboratory tests upon admission and patient's pharmacotherapy

Physical examination was within normal limits, apart from an increased BMI of 29.05 kg/m2; the patient was diagnosed as being overweight (weight 82 kg, height 168 cm). Laboratory tests were performed in accordance with the relevant standards, which showed the following findings: complete blood count (CBC), basic metabolic panel (BMP), liver function test (LFT), renal function panel (RFT), fasting lipid panel (FLP) were within norm, urinalysis (UA) showed iatrogenic glucosuria related to dapagliflozin pharmacotherapy; total iron binding capacity (TIBC), total iron (Fe), vitamin B12 (Vit B12), vitamin D3 (Vit D3), and folate tests were also within normal limit. Her current medication therapy was as follows: acetylsalicylic acid 75 mg, valsartan 80 mg, torasemide 5 mg, metoprolol 100 mg, nitrendipine 10 mg, chlortalidone 50 mg, doxazosine 8 mg, eplerenone 50 mg, dapagliflozin 10 mg, metformin 1,000 mg, atorvastatin 20 mg, pantoprazole 20 mg, clonazepam 1 mg, paroxetine 20 mg, fluoxetine 10 mg, mianserin 30 mg, estazolam 2 mg, and vitamin D 2,000 IU daily. Based on her interview and the previous treatment for MDD, the decision to perform PSG and psychiatric examination was made.

### 4.3. The polysomnography findings

The PSG equipment used to examine the patient was Nox-A1 (Nox Medical, Reykjavík, Iceland). The v-PSG evaluation was performed without adaptive night. The results of v-PSG were evaluated by sleep specialists according to the AASM (American Academy of Sleep Medicine). Full-night PSG recordings were divided into 30-s epochs and scored. PSG findings included the following: sleep latency; REM latency; TST; SE; and the duration of N1 (sleep stage 1), N2 (sleep stage 2), N3 (sleep stage 3), and REM. Polysomnograms were supplemented with all-night video and audio recordings in high resolution. Respiratory events were scored according to the AASM: the reduction of more than 90% of airflow for ≥10 s was scored as apnea, and a reduction of ≥30% for ≥10 s, with a ≥3% decline in blood oxygen saturation or followed by arousal, was scored as hypopnea.

#### 4.3.1. The PSG examination

**Figure 2:**
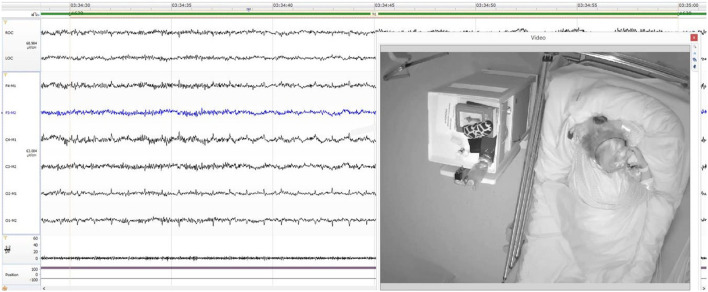
The patient during the N1 sleep stage holding an apple in her mouth.

During the PSG examination, the patient was diagnosed with mild obstructive sleep apnea (AHI=6.7/h), but in the video recording, an episode of nocturnal eating was observed. At the beginning, there was a transition from REM sleep to wakefulness, and the patient got up from the bed, reached for an apple from the table, and began to eat it. Then, she put the apple on the bedside table and went to the toilet. With the lights off, she walked from the toilet to her bed with her eyes closed and began searching for the apple by palpating the things on the table in the dark. After a few seconds, she found the apple, laid down in her bed, and began to eat it greedily. 5 min later, she stopped eating and just kept the apple in her mouth where the EEG showed theta waves and microsleep episodes. After a short break, she began to eat the apple again and in N1 sleep cycle, the patient holding the apple in her mouth the whole time. An image of the patient during this situation is presented in Figure 2. At the end, the core of the apple fell out of her mouth. The eating episode was covered by partial amnesia. The patient remembered the eating episode but did not remember the details. It is worth noting that the patient had food in her mouth in the N1 stage, which could be potentially dangerous due to the possibility of choking. Based on these findings, SRED was suspected. The other parameters measured by PSG are presented in Table 3. The patient was discharged from the ward in good condition, with recommendations of reducing her body weight and avoiding sleeping on her back.

**Table 3:** Polysomnography results of the case report presented.

**Polysomnography parameters**	**The polysomnography examination**
Episode of nocturnal eating	Present
Total sleep time (TST)	405.0 min
N1 sleep stage	5.3% of TST
N2 sleep stage	57.2% of TST
N3 sleep stage	6.90% of TST
Rapid eye movement (REM)	30.60% of TST
Sleep latency	19.8 min
REM latency	133.5 min
Sleep efficiency	86.2%
Snoring	36.2% of TST
Arousal index	1.80 events/h
Apnea–Hypopnea index	6.7 events/h
Oxygen desaturation index	5.9 events/h
Respiratory disturbances index	6.7 events/h
Mean saturation O2	91.90%
Minimal saturation O2	74.00%
O_2_ saturation < 90%	23.10%
Mean desaturation drop	4.50%
Wake after sleep onset	44.5 min
Periodic limb movement in sleep index	0 events/h
Movement	7.60% of TST
Mean heart rate	70.90 beats/min
Minimal heart rate	66.00 beats/min
Maximal heart rate	80.00 beats/min

### 4.4. Psychiatric consultation

A 66-years-old married female were invited to psychiatric evaluation. Her past medical history also consisted of psychiatric disorder—major depressive disorder (MDD). She was diagnosed with MDD and insomnia at the age of 49 years. For the most of the time, she was being treated with pharmacotherapy and was hospitalized thrice in an inpatient ward (last stay in 2009). She reported many suicidal attempts, the first one at the age of 22 and the most recent one 5 years ago.

In the past, the patient was treated with several antidepressive and anxiolytic agents. At the time of the enrollment to the study and v-PSG examination, she was being treated with paroxetine 20 mg daily, fluoxetine 10 mg daily, mianserin 30 mg daily, and clonazepam 1 mg in the morning and estazolam 2 mg at night (before sleep). This medication was followed for the past 4 years except for clonazepam, which was introduced 1 year ago, and fluoxetine, which was reduced from 20 mg down to 10 mg daily in mid-2022. She was reporting increased appetite and problems with the maintenance of her body weight to her psychiatrist, but the changes in pharmacotherapy did not address these problems. According to the patient, SRED episodes started around 2 years prior, but they happened rarely. Their frequency and intensity increased dramatically around 1 year ago, when clonazepam was introduced. At the time of the examination, she was in the remission of depressive symptoms. However, she reported that she cannot withdraw clonazepam in the morning because without it she became extremely anxious, angry, and irritable, and she could not fall asleep without estazolam in the evening. The interview and examination revealed that she has developed a dependence on benzodiazepine agents.

As the present pharmacological treatment could have led to drug-drug interaction with probably presentation e.g., a significant increase in appetite and the number of SRED episodes, to reduce the symptoms and avoid the interactions, changes were introduced. Firstly, paroxetine was withdrawn, and instead, fluoxetine dosage was increased up to 30 mg. Secondly, a slow reduction in clonazepam and estazolam was recommended, and low-dose (150 mg) pregabalin was introduced to reduce anxiety. The dose of mianserin dose was reduced with a plan of total withdrawal.

## 5. Discussion

In this systematic review, the available literature on SRED patients was assessed, in particular their PSG findings and video-recorded sleep behavior. Moreover, this review aimed to investigate whether there was any relationship between deviations in PSG findings and the presence of SRED. Although the majority of the studies reported a moderate or high risk of bias, this review may allow us to draw several conclusions.

Firstly, the prevalence of SRED is higher in women. This is also the case in our review, in which the female gender predominated among included studies, where there were 130 women of 206 patients (63.01%). Furthermore, SRED was found in young people in their mid-20 s, whereas most studies reported on 30-year-olds (6). This syndrome can develop at any age, e.g., in a 9-year-old boy (19) or, as in the present case, a 66-year-old woman. While examining comorbidities with NREM parasomnia or those predisposing to SRED, 9 studies indicated the presence of these, as suggested in the available literature (6), sleepwalking was the most common comorbidity disease to SRED among included papers. In comparison to the general population, the prevalence of sleepwalking among SRED patients is higher (23.9%) than healthy people (1.5%) (31). However, obstructive sleep apnea and periodic legs movement syndrome were less common in contrast to the general population (8.73% vs. 28.6%) and (5.83% vs. 7.6%) (32, 33). Restless leg syndrome and sleep terrors were presented within the normal range (3.0% to 15% for RLS, 1.0% to 2.6% for sleep terrors) (34, 35). In the present case, it was obstructive sleep apnea (OSA).

In accordance with the NREM definition, parasomnia presents with awakening from sleep or the behavior typical of the presented syndrome in the deep sleep stage, i.e., most often in the N3 stage (4). This review revealed quite large differences in this area. The literature rarely describes an episode of nocturnal eating during PSG, which may be due to several causes. Firstly, nocturnal eating in SRED may not occur every night depending on the duration of the disease and the patient. The literature describes from 1 episode per week to as many as 10 episodes per night (36). Furthermore, changing the environment can be stressful for the patient, and a night spent in an unfamiliar hospital environment can result in enough sleep to prevent SRED behavior (7). Finally, the lack of food in the patient's room should also be considered. Following the recording of the episode, 95 episodes of eating occurred in 57 patients among gathered 206 people, therefore prevalence of episodes is 27.77%. But they should occur in the N3 sleep stage, as mentioned earlier. However, in 9 articles reporting these episodes, only 30% (29 from 95 episodes) were in the N3 stage. Vertrugno et al. (25) reported only the average number of eating episodes; however, it is not known at which point in the sleep they occurred, therefore it was not included in these calculations. As shown, the N3 stage was in the minority. In the present case report, the patient awoke from REM sleep and experienced an episode of eating while awake. The partial or complete loss of awareness of eating is one of the criteria for the diagnosis of SRED, and the majority included studies that met this criteria. However, in one study (25), all participants diagnosed with SRED were fully aware of what was happening at night and in Winkelman (24) 2 from 23 patients also had consciousness during the episode of eating, which is a rather unusual and typical characteristic of NES.

In Table 2, the PSG parameters measured during sleep studies as part of individual studies are presented. Unfortunately, this review has some limitations. Due to the large discrepancy in the years of studies and the lack of appropriate equipment and standardization of the results, not all records include the selected values, and in some studies, the authors provide only abnormal results. According to Hertenstein et al. (37), PSG parameters may vary depending on age, sex, and habituation to a particular environment as PSG findings varied based on the number of nights spent in the sleep laboratory. The mean reference ranges for PSG results came from Hertenstein et al. study (37) and based on the analysis of these data and values shown in Table 2, it seems that most of the measurements of the characteristics are within this norm. But some of them are reduced, such as the N3 sleep stage in the group of 10 participants in Komada et al. (29), or increased, such as the AHI in Vetrugno et al. (24) or PLMSI in Schenck et al. (27); however, these differences may be attributable to comorbid sleep disorders or medications. Patients diagnosed with SRED using PSG do not show altered parameters specific to this syndrome; in fact, when an eating episode was observed during the examination, the parameters were still normal or the given values were different in each study. Not surprisingly, some authors have not distinguished between SRED and NES and described these disorders in one category (38, 39). In order to eliminate the effect of “first night” during hospital stay on PSG results, portable PSG e.g., headbands should be considered to use (40). These devices have similar accuracy in monitoring sleep parameters like PSG, however, also have some limitations in detecting state of wakefulness (41).

A sleep disorder similar to SRED characterized by nocturnal eating and sometimes causing problems in making diagnose is NES. NES involves episodes of consuming at least 25% of daily calorie intake during night with remaining consciousness, which is the complete opposite to SRED. As a consequence of NES, the patient experiences morning anorexia, daily dysfunction, decreased mood and other features from Table 1. The disorder has not been included in ISCD-3 and has recently been classified in the American Psychiatric Association's Diagnostic and Statistical Manual of Mental Disorders (DSM-5) (11). In spite of the obvious differences in symptoms between SRED and NES, sometimes are resulting in diagnostic problems if a thorough anamnesis, nutritional questionnaires, and PSG are not applied. PSG allows us to check the level of awareness during an eating episode, which can be conclusive for the diagnosis (13). Additionally, Schenck et al. (27) indicated, that SRED itself is already a heterogeneous disease with many causes and polysomnography is crucial to made correct diagnosis and select appropriate treatment, similar results were received in Winkelman study (24). These conclusions were drawn before diagnostic criteria for sleep-related eating disorder were established in ISCD, however, nowadays in ISCD-3 PSG was considered as not necessary to confirm this disease.

In light of the presented case and systematic report, it is clear that this woman with coexisting MDD and benzodiazepine drug dependence has iatrogenic SRED induced by clonazepam. As mentioned in World Health Organization (WHO) databases, clonazepam is estimated as a cause of SRED in about 1.3% of cases (8). Unfortunately, reports of this condition in WHO databases are based on patient history, reported by anyone to this register. In the literature, one confirmed case of SRED induced by clonazepam with full medical examination is present, enriched by PSG records (19). Based on the findings of the present case report, the observed metabolic disturbances and sleep-related eating could be linked to pharmacotherapy. The patient presented changes in the parameters during PSG, during the PSG examination; however, this could be a result of OSA.

The present review focused on evaluating the PSG findings of SRED patients. Unfortunately, this review has limitations and drawbacks. First of all, most of the studies included in our review contained data obtained from studies with a large sample size, which contained inaccurate conclusions and from the description of single cases, thus receiving high imprecision according to the risk of bias assessment tools. Based on this review, it can be emphasized that there is a need for further intensive research into the pathophysiology of SRED and a better understanding of it, as well as developing objective methods that will reliably and independently contribute to the appropriate diagnosis and initiation of effective treatment. Future studies using v-PSG on a large group of patients may improve the diagnostic evaluation and uncover new characteristics associated with SRED. In the future to made a correct diagnosis may help modern technology consist of e.g., portable cameras. At the time these devices could be used in patient houses to reduce the influence of hospital stay on PSG results. Currently, however, the diagnosis is based on a thorough clinical interview, but PSG can be a valuable additional tool for clinicians.

## 6. Conclusion

Polysomnography is not necessary for the diagnosis of SRED. However, it could facilitate the diagnosis and differentiation of SRED from other eating disorders. PSG also has limitations in capturing eating episodes and additionally, its cost effectiveness should be considered in the diagnostic process. More original studies into the causes and pathophysiology of SRED are needed with a potential low risk of bias because classifying SRED as NREM parasomnias can be inappropriate as it does not always occur during deep sleep. In the present case report, the patient took drugs to treat multiple diseases, and they could have side effects. SRED may lead to choking and obesity, which contributes to the occurrence and difficulty in the treatment of primary diseases such as diabetes, atherosclerosis, hypertension, and cardiovascular diseases.

## Data availability statement

The raw data supporting the conclusions of this article will be made available by the authors, without undue reservation.

## Author contributions

HM was involved in the conception, visualization, and supervision of the study. BB, TW, and MM-Z collected the data and wrote the manuscript. MM-Z and HM examined the patient. TW conducted the psychiatric consultation. MW and GM were involved in the revision of final version of the manuscript. All authors have agreed to the published version of the manuscript.
