# Supplementary material for: Polysomnography findings in sleep-related eating disorder: a systematic review and case report
Source: Front Psychiatry. 2023 May 10;14:1139670. doi: 10.3389/fpsyt.2023.1139670 (PMC10206059; doi:10.3389/fpsyt.2023.1139670)
Supplement: Supplementary file 1 [file Data_Sheet_1.PDF]

Table 3. Risk of bias assessment following the Joanna Briggs Institute (JBI) checklist for the 6 included case reports.

Study	Questions from the JBI checklist for case reports								Overall evaluation of risk of bias
	1. Were the patient's demographic characteristics clearly described?	2. Was the patient's history clearly described and presented as a timeline?	3. Was the current clinical condition of the patient on presentation clearly described?	4. Were diagnostic tests or assessment methods and the results clearly described?	5. Was the intervention(s) or treatment procedure(s) clearly described?	6. Was the postintervention clinical condition clearly described?	7. Were adverse events (harms) or unanticipated events identified and described?	8. Does the case report provide takeaway lessons?	
(30)	Yes	Yes	Yes	No	Yes	Yes	Unclear	Unclear	Moderate
(31)	No	No	No	Unclear	Yes	No	Yes	Unclear	High
(7)	No	Yes	Yes	Yes	Yes	No	No	Yes	Moderate
(32)	Yes	Yes	Yes	No	Yes	Yes	No	Unclear	Moderate
(14)	No	Yes	Yes	Yes	Yes	Yes	No	Unclear	Moderate
(33)	No	Yes	Yes	Yes	Yes	Yes	Yes	Unclear	Moderate

Table 4. Risk of bias assessment for the seven included descriptive studies following the Joanna Briggs Institute (JBI) checklist

Study	Questions from the JBI checklist for descriptive studies										Overall evaluation of risk of bias
	1. Is there congruity between the stated philosophical perspective and the research methodology?	2. Is there congruity between the research methodology and the research question or objectives?	3. Is there congruity between the research methodology and the methods used to collect data?	4. Is there congruity between the research methodology and the representation and analysis of data?	5. Is there congruity between the research methodology and the interpretation of results?	6. Is there a statement locating the researcher culturally or theoretically?	7. Is the influence of the researcher on the research, and vice versa, addressed?	8. Are participants, and their voices, adequately represented?	9. Is the research ethical according to the current criteria or, for recent studies, is there evidence of ethical approval by an appropriate body?	10. Do the conclusions drawn in the research report flow from the analysis, or interpretation, of the data?	
(13)	Yes	Yes	Yes	Yes	Yes	No	Yes	Yes	No	Yes	Low
(39)	Yes	Yes	Yes	Yes	Yes	No	No	Yes	No	Yes	Moderate
(37)	Yes	Unclear	Yes	Yes	Yes	No	No	Yes	No	Yes	Moderate
(38)	Yes	No	Yes	Yes	Yes	No	Unclear	Yes	Yes	Yes	Moderate
(36)	Yes	Yes	Yes	Yes	Yes	No	No	Yes	No	Yes	Moderate
(35)	No	Yes	Yes	Yes	Yes	No	No	Yes	No	Yes	Moderate
(34)	No	Yes	Yes	Yes	Yes	No	No	Yes	No	Yes	Moderate

Table 5. Assessment risk of bias in the case–control and cohort study using ROBINS-I tools.

Study	Domains checked by ROBINS-I tool							Overall evaluation of risk of bias
	1. Bias due to confounding	2. Bias in selection of participants in the study	3. Bias in classification of interventions	4. Bias due to deviations from intended interventions	5. Bias due to missing data	6. Bias in measurement of outcomes	7. Bias in selection of the reported result	
(40)	Moderate	Low	Low	Moderate	Serious	Low	Low	Serious
(41)	Low	Low	Low	Moderate	Moderate	Low	Low	Moderate
